# Reproducibility of Methods to Detect Differentially Expressed Genes from Single-Cell RNA Sequencing

**DOI:** 10.3389/fgene.2019.01331

**Published:** 2020-01-17

**Authors:** Tian Mou, Wenjiang Deng, Fengyun Gu, Yudi Pawitan, Trung Nghia Vu

**Affiliations:** ^1^ Department of Medical Epidemiology and Biostatistics, Karolinska Institutet, Stockholm, Sweden; ^2^ School of Mathematical Sciences, University College Cork, Cork, Ireland

**Keywords:** single cell, RNA sequencing, differential expression, rediscovery rate, comparison

## Abstract

Detection of differentially expressed genes is a common task in single-cell RNA-seq (scRNA-seq) studies. Various methods based on both bulk-cell and single-cell approaches are in current use. Due to the unique distributional characteristics of single-cell data, it is important to compare these methods with rigorous statistical assessments. In this study, we assess the reproducibility of 9 tools for differential expression analysis in scRNA-seq data. These tools include four methods originally designed for scRNA-seq data, three popular methods originally developed for bulk-cell RNA-seq data but have been applied in scRNA-seq analysis, and two general statistical tests. Instead of comparing the performance across all genes, we compare the methods in terms of the rediscovery rates (RDRs) of top-ranked genes, separately for highly and lowly expressed genes. Three real and one simulated scRNA-seq data sets are used for the comparisons. The results indicate that some widely used methods, such as edgeR and monocle, have worse RDR performances compared to the other methods, especially for the top-ranked genes. For highly expressed genes, many bulk-cell–based methods can perform similarly to the methods designed for scRNA-seq data. But for the lowly expressed genes performance varies substantially; edgeR and monocle are too liberal and have poor control of false positives, while DESeq2 is too conservative and consequently loses sensitivity compared to the other methods. BPSC, Limma, DEsingle, MAST, t-test and Wilcoxon have similar performances in the real data sets. Overall, the scRNA-seq based method BPSC performs well against the other methods, particularly when there is a sufficient number of cells.

## Introduction

Traditional gene expression profiling with high-throughput RNA-sequencing technology measures the aggregated expression levels of genes from a collection of millions of cells. Such bulk-cell RNA-sequencing cannot capture cellular heterogeneity since there is no cell-specific information (Miao and Zhang, 2016; Jaakkola et al., 2017). Single-cell RNA sequencing (scRNA-seq) has developed rapidly as a powerful technology for studying transcriptomics at the single-cell level (Sandberg, 2014). However, compared to bulk-cell data, scRNA-seq data has a higher level of noise due to both biological and technical reasons, for example, lower input materials, cell-cycle phase, amplification biases, and the so-called dropout and bursting events (Dal Molin et al., 2017; Jaakkola et al., 2017; Soneson and Robinson, 2018). Such events are caused by the stochastic nature of the gene expression process at the single-cell level (Gong et al., 2018). The dropout events generate zero expression, statistically leading to zero inflation in the gene-expression distribution at a much higher proportion than expected under the standard negative-binomial model commonly assumed in bulk-cell data (Miao et al., 2018). Aggregation of expression in bulk-cell data reduces the effects of these single-cell events.

Differential expression (DE) analysis to discover quantitative changes between different groups or conditions plays an important role for understanding the molecular basis of phenotypic variation. However, due to the unique characteristics of scRNA-seq data, it is not immediately obvious that we can just use standard methods developed for bulk-cell data. A particular challenge is dealing with the large number of low (or zero) read counts in the scRNA-seq data. A previous study (Love et al., 2014) has shown the phenomenon that weakly expressed genes tend to produce more differences than highly expressed genes. For instance, to tackle this issue, several DE methods have been developed for scRNA-seq data, for example, BPSC (Vu et al., 2016), MAST (Finak et al., 2015), and monocle (Qiu et al., 2017). In general, bulk-cell–based DE methods were not originally designed to deal with a large fraction of lowly expressed genes. Yet, in practice, many studies use the bulk-cell−based DE methods for single-cell data, such as edgeR (Wang et al., 2016) or limma (Ziegenhain et al., 2017). Furthermore, various pipelines and workflows of RNA-seq analysis do not consider scRNA-seq data specifically (Lun et al., 2016; Chen et al., 2016; Law et al., 2016) and suggest users apply the bulk-cell−based methods to scRNA-seq data (Zhu et al., 2017).

These bulk-cell−based methods are methodologically sophisticated, and they have been used for scRNA-seq data, but evaluation of their applicability to scRNA-seq data is still uncommon and different studies have reported opposite results. For example, authors in a recent study (Jaakkola et al., 2017) compared five DE methods, including two single-cell–based methods and three bulk-cell−based methods. They concluded that the original DESeq (Anders and Huber, 2010) and limma (Law et al., 2014) are not suitable for scRNA-seq data. In contrast, another comparative study (Miao and Zhang, 2016) declared that DESeq tends to outperform other methods on scRNA-seq data. Most comparative studies (Miao and Zhang, 2016; Dal Molin et al., 2017; Jaakkola et al., 2017; Soneson and Robinson, 2018) agree that bulk-cell–based methods are applicable to scRNA-seq even though there is a lack of agreement in finding DE genes by these DE methods (Wang et al., 2019) and it is difficult to identify the best performing tool for DE analysis of scRNA-seq data (Dal Molin et al., 2017). Therefore, further evaluations of these DE methods, including both bulk-cell– and single-cell–based methods in different aspects, are warranted for better understanding of the methodologies when applied to scRNA-seq studies.

To compare the DE methods, previous studies have used conventional statistics such as type-I error rate, false discovery rate (FDR) and receiver operating characteristic (ROC) curve. Notably, these metrics are applied to the full collection of genes. Reproducibility is also an important metric, although it is sometimes calculated differently in the different studies. For example, a recent study (Miao and Zhang, 2016) assesses the reproducibility of the methods by looking at the average of the overlap of top 1,000 DE genes (ranked by p-value) across 20 replicates. In each replicate, a control group and a testing group are sampled with a different random seed. Another measure of reproducibility (Jaakkola et al., 2017) compares the precision and recall of the detection of all DE genes between the full data set and its subsets.

In this study, we compare the performance of nine DE methods, including both bulk-cell and single-cell–based approaches as well as general statistical tests not specifically designed for RNA-seq data. We focus on the reproducibility of the methods in terms of rediscovery rate (RDR) (Ganna et al., 2014) of top-ranking genes. RDR is defined as the proportion of top-ranking findings detected from a training sample that are replicated in a validation sample. In high-throughput studies, the RDR is determined by both the false positive rate (FPR) and power (Ganna et al., 2014), so it is a convenient and easily understood metric for the comparison of methods. Limiting the assessment to top-ranking genes turns out to be important. Firstly, it follows the data analytic process we perform in practice, where the top-ranked genes are usually considered the most interesting ones for further biological analyses or interpretation. Secondly, some methods perform differently for the top-ranked genes and across all genes. Besides the RDR, type-I error rate or FPR, and ROC are also used as extra metrics for the comparisons.

To get realistic distributional characteristics and capture some diversity in single-data data, we utilize three real scRNA-seq data sets; in addition, we use simulated data from the beta-Poisson model (BPSC), which has been suggested for scRNA-seq data in a recent study (Vu et al., 2016). Because of their distinct distributions, the groups of highly and lowly expressed genes are also considered separately, as the latter is more affected by single-cell specific events such as dropouts.

## Results

We compare nine methods for detecting differentially expressed isoforms, including edgeR (Robinson et al., 2010), DESeq2 (Love et al., 2014), DEsingle (Miao et al., 2018), monocle (Qiu et al., 2017), BPSC (Vu et al., 2016), MAST (Finak et al., 2015), t-test (Welch, 1947), Wilcoxon rank sum test (Hollander et al., 2013), limmatrend (Law et al., 2014). Among those, edgeR, DESeq2 and limmatrend are designed for bulk-cell RNA-seq analysis; and DEsingle, monocle, BPSC, and MAST are developed based on scRNA-seq data. T-test and Wilcoxon rank-sum test are general comparison tests not specific to RNA-seq data. **Table 1** compares the methods in terms of (i) distribution assumption, (ii) original data motivation (bulk-cell or single-cell data), (iii) test statistic, and (iv) run time for a typical data set used in the comparisons. We also state the exact version of each software tool used in the comparisons.

**Table 1:** List of the differential expression analysis methods.

Method	Distribution assumption	Designed for	Test statistic	Run time	Version [Ref.]	Input
BPSC	Beta-Poisson	Single cell	z-test	Hours	0.99.2 (Vu et al., 2016)	CPM
DEsingle	Zero-Inflated Negative Binomial	Single cell	Likelihood ratio test	Hours	1.2.1 (Miao et al., 2018)	raw counts
MAST	Normal (Generalized linear hurdle)	Single cell	Likelihood ratio test	Minutes	1.8.2 (Finak et al., 2015)	log_2_(CPM+1)
monocle	Normal (Generalized additive model)	Single cell	Likelihood ratio test	Minutes	2.10.1(Qiu et al., 2017)	raw counts
DESeq2	Negative Binomial	Bulk cell	Wald test	Minutes	1.22.2 (Love et al., 2014)	raw counts
edgeR	Negative Binomial	Bulk cell	Quasi-likelihood F-test	Minutes	3.24.3 (Robinson et al., 2010)	raw counts
limmatrend	Normal (linear model)	Bulk cell	Empirical-Bayes Moderated t-statistics	Seconds	(Law et al., 2014)	log_2_(CPM+1)
t-test	Normal	General	t-test	Seconds	(Welch, 1947)	log_2_(CPM+1)
Wilcoxon	Nonparametric	General	Wilcoxon	Minutes	(Hollander et al., 2013)	log_2_(CPM+1)

To get realistic distributional characteristics, the following three real scRNA-seq data sets are used as the basis for simulations. (Different papers and projects use isoform- and gene-level expressions. For simplicity, we shall use the terms “isoform” and “gene” interchangeably.)

- Breast-cancer cell line MDA-MB-231 data set (Athreya et al., 2017) has two groups: control and metformin-treated, 80 cells in each group. The expression estimates of 26,775 isoforms from Cufflinks are used in the analysis.
- Mouse embryonic stem cells (mESCs) belong to two groups from different culture conditions, 94 cells in group 1 and 174 cells in group 2; see the Materials and Methods section for details. The expression estimates of 112,593 isoforms are provided by the *Conquer* project (Soneson and Robinson, 2018).
- Neuronal progenitor cells (NPCs) also form two groups, one from the patient and the other from a healthy donor (Iacono et al., 2018), 360 cells in each group. The expression estimates of 41,020 genes are provided by the bigSCale project (Iacono et al., 2018).

In addition, we also simulate single-cell data based on the beta-Poisson model (Vu et al., 2016). The variation in sample sizes of the three real data sets, from 160 to 720, allows us to compare the performance of each method at different sample sizes. More details of the methods and data sets are given in the Materials and Methods section.

In each experiment, the comparison focuses on the DE analysis of two predefined groups of cells. Briefly, an equal number of samples is randomly selected from the two groups in the original data set to generate the training set. For each sampled cell from a real data set, all isoforms are taken together; this preserves the statistical dependencies between the isoforms. For the validation set, a different set of samples from both groups is selected. The selection of training and validation sets is repeated 50 times to average out the effect of random selection. Note that the training and validation sets are always disjoint. The nine DE methods are then applied to the training and validation sets separately.

### Type-I Error Control

For each real data set, we generate a null data set by randomly sampling from the two groups combined (i.e., ignoring the group labels). Thus, the null data sets are expected to have no true DE isoforms, and the p-value distribution of each method is expected to be uniform. Theoretically, the p-values should follow a uniform distribution if the null hypothesis is true (Murdoch et al., 2008; Bland, 2013). The uniformity of p-value distribution under the null hypothesis can be used to assess the performance of methods. We calculate the type-I error rate by recording the fraction of the detected DE isoforms that are assigned a significant p-value (*p* < 0.05). This fraction is also known as the FPR. To highlight the effects of the dropout events, which tend to produce low expression and zero inflation, we split the isoforms into two groups based on the expression level: highly expressed isoforms and lowly expressed isoforms. The former refers to the isoforms with an estimated expression above 1 transcripts-per-million (TPM) in more than 25% of the cells, and the remaining isoforms are assigned to the latter. This threshold was also suggested in a recent comparative study of DE methods in scRNA-seq (Soneson and Robinson, 2018).

**Figure 1:**
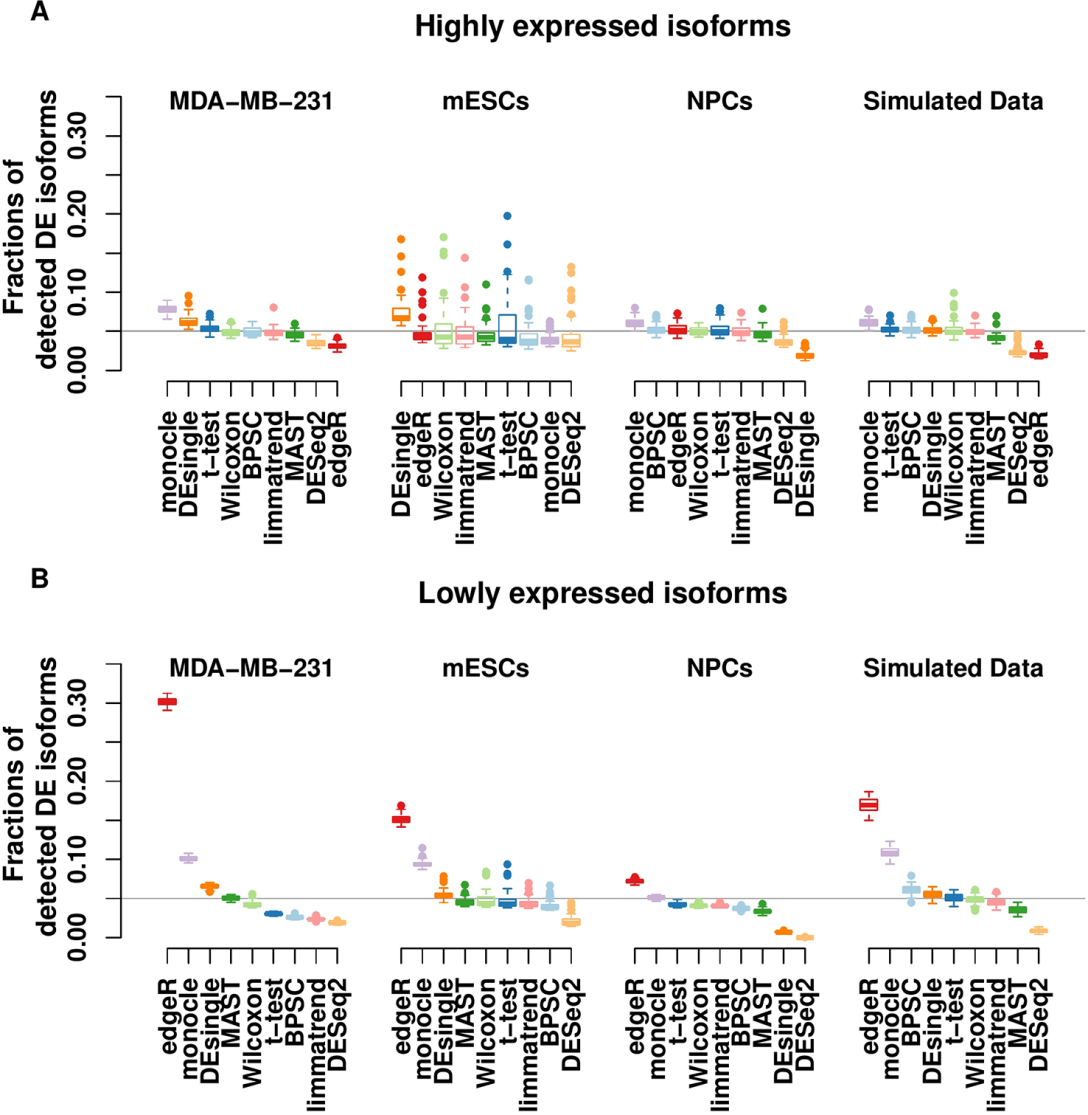
Type-I error control for the groups of highly expressed isoforms **(A)** and lowly expressed isoforms **(B)** of the three real scRNA-seq data sets and the simulated data set. The values in the y-axis are the fractions of isoforms with *p* < 0.05 under the null hypothesis. The horizontal line indicates the expected error rate at 0.05. Box plots of the methods in the x-axis are the collection from 50 replicates. The methods are ordered by median false positive rate (FPR) across all replicates. The number of highly expressed isoforms in MDA-MB-231, mESCs, NPCs and simulated data sets are 8,299, 31,895, 10,422, and 8077, respectively. The corresponding number of lowly expressed isoforms are 18,476, 80,698, 30,378, and 1,923.

Results in **Figure 1A** show that for highly expressed isoforms, most methods manage to control the FPR close to the target 0.05. Two single-cell–based methods, monocle and DEsingle, are not stable, as their FPRs fluctuate the most from the expected error rate. As expected, the bulk-cell–based methods, edgeR, DESeq2, and limmatrend, perform well on this group, and DESeq2 is the most conservative.

For the lowly expressed isoforms, DESeq2 is also the most conservative method, **Figure 1B**. It identifies fewer significant isoforms, so the FPR is significantly lower than the expected level (0.05) in all data sets. In contrast, edgeR has the highest FPR, sometimes substantially above the target value. Similarly, monocle also has a large number of false positive findings. The FPR of DEsingle has a slight variation, as it is liberal for MDA-MB-231 data set, conservative for NPCs data set, and performs rather well in the other data sets. Thus, it seems the performance of DEsingle is not stable and highly dependent on data sets. The histograms of p-values (**Figure S1** in the Supplementary report) further illustrate that few methods returned uniformly distributed p-values under the null hypothesis for the lowly expressed isoforms, while most methods have a better uniformity for the highly expressed isoforms.

### The RDR

The RDR is the proportion of the top-ranking DE isoforms in the training set that is found to be significant (*p* < 0.05) in the validation set. The RDR is calculated based on the top 5%, 10%, 20% DE and all isoforms in the training set.

#### RDR Analysis Under the Null Hypothesis

**Figure 2:**
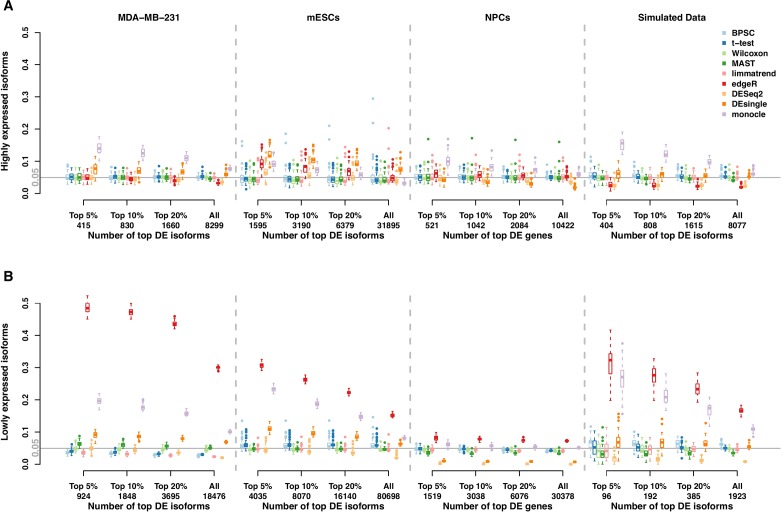
Rediscovery rate (RDR) of differential expression (DE) isoforms in the real and simulated scRNA-seq data sets under the null hypothesis calculated from the top 5%, 10%, 20% DE and all isoforms. (Panels **A** and **B**) present the results of groups of highly expressed isoforms and lowly expressed isoforms, respectively. The number of highly expressed isoforms in MDA-MB-231, mESCs, neuronal progenitor cells (NPCs), and simulated data sets are 8,299, 31,895, 10,422, and 8,077, respectively. The corresponding number of lowly expressed isoforms are 18,476, 80,698, 30,378, and 1,923.

The RDR of the null data sets from the real data in Section 2.1 are reported in **Figure 2**. Panels A and B present the results for the groups of highly expressed isoforms and lowly expressed isoforms, respectively. Under the null hypothesis of no group effect, the expected RDR is 0.05. Similar to the results from the type-I error control in Section 2.1, the RDRs of all methods are generally better for highly expressed isoforms. Monocle and DEsingle are the worst, as their RDRs are often far from 0.05. However, the performances improve for the larger number top DE isoforms. For example, the RDR of monocle for all isoforms in the NPCs data set is very close to the expected value, but it is much higher than 0.05 among the top 5% DE isoforms. Similarly, for the mESCs data set, the RDR of edgeR for all isoforms is close to 0.05, but it is consistently higher than this target value for the smaller number of top DE isoforms. Thus, comparing the performances based on all isoforms could be misleading.

These patterns are much more pronounced for lowly expressed isoforms; see **Figure 2B**. In this case, edgeR performs worst in all data sets; this result is consistent with other studies (Soneson and Robinson, 2018). The performances of DESeq2 still tend to be conservative in both groups of isoforms, while other methods generally have RDR around the expected value.

We further evaluate RDR of the DE methods in the simulated beta-Poisson data set. Results from 50 replicates of the null data sets from the simulated data are reported in the rightmost plots of **Figures 2A**, **B**. The similar patterns of RDR of DE methods for both isoform groups confirm the results from the real data sets. In particular, monocle has poor performances in both groups, and edgeR does not perform well with lowly expressed isoforms.

#### RDR Analysis Under the Alternative Hypothesis

**Figure 3:**
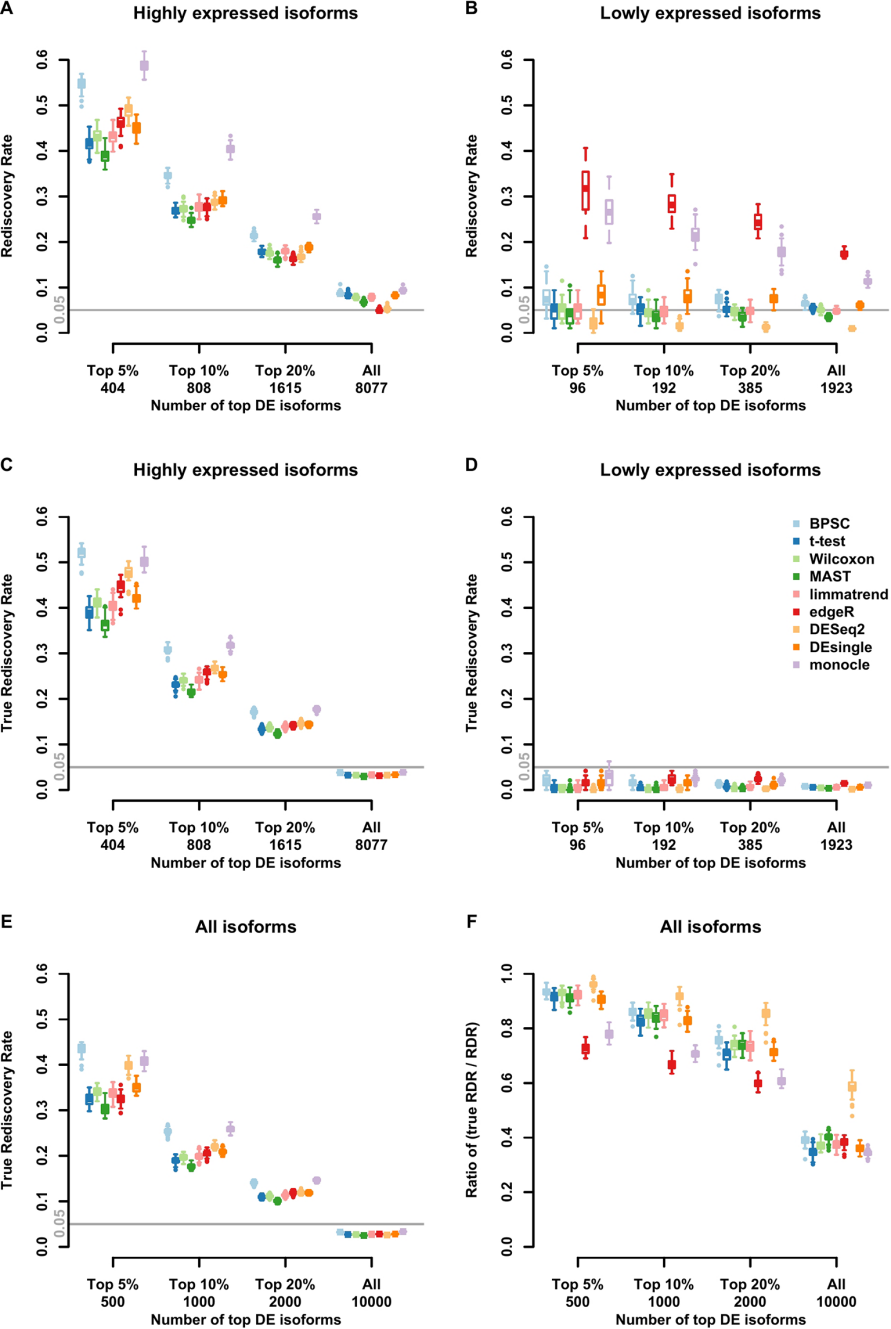
Observed rediscovery rate (RDR) and true rediscovery rate (TrueRDR) of differential expression (DE) isoforms in the simulated beta-Poisson data set under the alternative hypotheses calculated among the top 5%, 10%, 20% DE and all isoforms. (Panels **A** and **B**) present the rediscovery rate in the groups of highly and lowly expressed isoforms, respectively. (Panels **C** and **D**) display the true rediscovery rate collected from highly and lowly expressed isoforms separately. (Panels **E**) displays the true rediscovery rate collected from both highly and lowly expressed isoforms. (Panels **F**) presents the ratio between true RDR and observed RDR. The number of highly expressed isoforms in the simulated data set is 8,077, and the number of lowly expressed isoforms is 1,923.

Results of RDR analysis for the simulated beta-Poisson data under the alternative hypothesis are presented in **Figure 3**. As described in more detail in the Materials and Methods section, 5% of the isoforms are randomly selected to be differentially expressed between the two groups (hence true DE isoforms). For highly expressed isoforms (**Figure 3A**), monocle and BPSC have the highest RDRs across the top 5%, 10%, 20% and all DE isoforms, while edgeR is comparable to the rest. DESeq2 is conservative for the null data sets and the group of lowly expressed isoforms, but its performance is comparable to other methods. For lowly expressed isoforms, edgeR and monocle produce the highest RDRs compared to other methods (**Figure 3B**). However, remember that from the previous subsection we know these two methods have high false positive rates.

In the simulated data, we in fact know the true DE status, so we can evaluate the true RDR, which is defined as the proportion of the true positives in the validation set among the top DE isoforms identified in the training set. In other words, the true RDR is the intersection of rediscovered genes and true DE genes. This is shown in **Figures 3C**, **D**. First, let us consider panel D. While there are 5% true DE isoforms, the statistical power for the lowly expressed isoforms is tiny, so very few of the true DE isoforms appear among the top-ranking genes *and* these isoforms do not produce significant p-values in the validation set. Hence the rediscoveries are mostly false positives. This means that there are reproducible features of the data, such as zero inflation, that consistently create problems for monocle and edgeR to the point of producing false positives in validation data. These results highlight the challenge in finding true DE among lowly expressed isoforms, or equivalently, the ease of producing false positives.

From **Figure 3C**, the true RDRs of 3 methods including BPSC, monocle and DESeq2 are better than the other methods. The overall true RDRs are given in **Figure 3E**, which in this case look similar to the result for highly expressed isoforms, but do not reflect the results for lowly expressed ones. **Figure 3F** shows the ratio of true RDR to observed (RDR). DESeq2 has the highest ratio among the comparing methods, indicating a good specificity in detecting DE isoforms. However, DEseq2 generally discovers fewer true DE isoforms, i.e., lower sensitivity, compared to BPSC. Two methods of edgeR and monocle have a lower ratio than the other methods since they have more false discoveries. In the next section, the balance between sensitivity and specificity of the methods are taken into account *via* the ROC curve.

For the real data sets, there are no significant differences in RDR performance for the top 5%, 10%, 20% DE isoforms between nine DE methods in the group of highly expressed isoforms (**Figure S2A** in the Supplementary report). However, similar to the results of the simulated data set, RDRs of edgeR and monocle are highly liberal, while DESeq2 tends to be too conservative for the lowly expressed isoforms (**Figure S2B**). We have performed other simulations and analyzed two other datasets that confirmed this observation. This is given in the Supplementary Material and described in the Discussion section.

### ROC Performance

**Figure 4:**
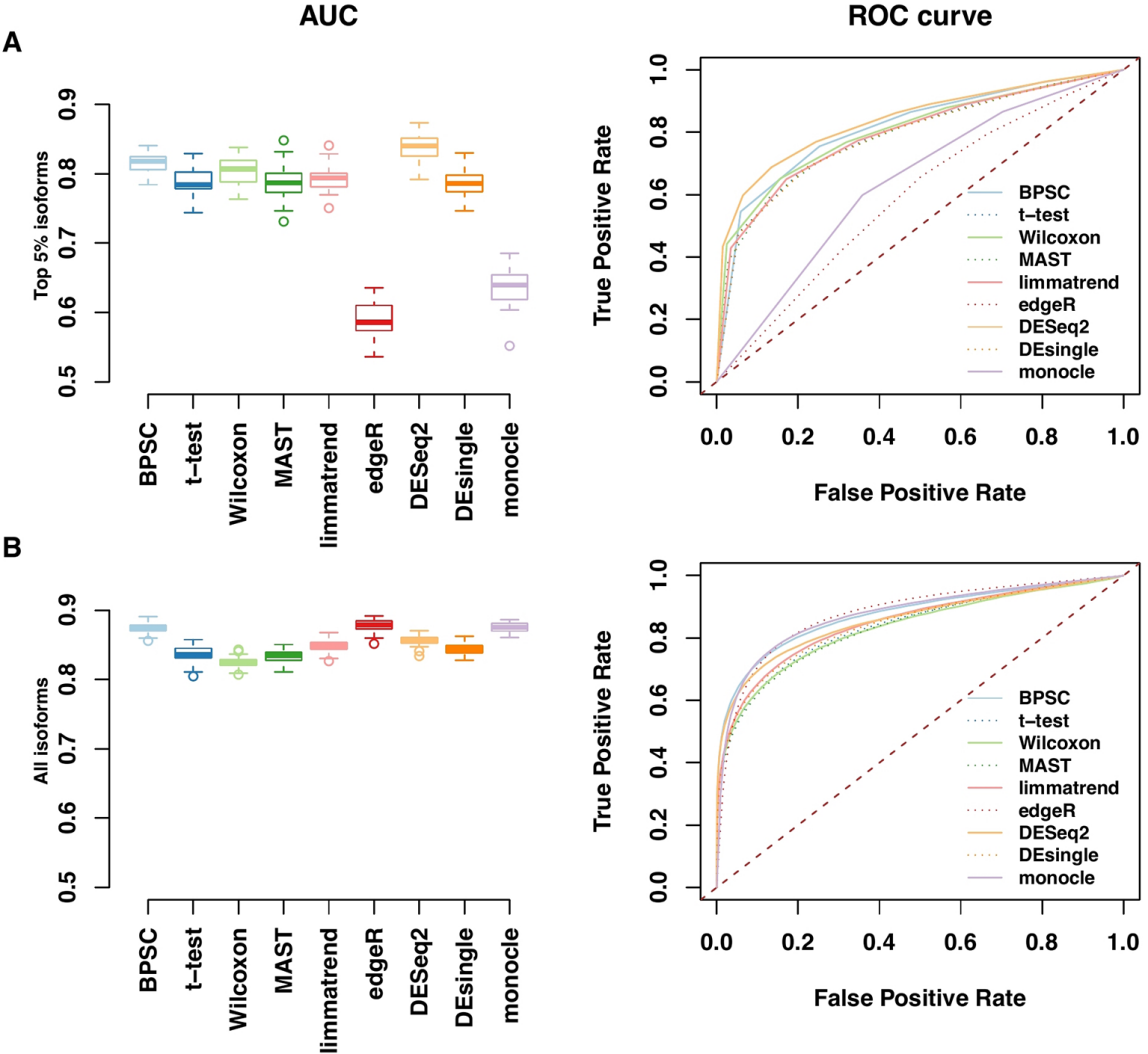
Receiver operating characteristic (ROC) and AUC performances for top 5% DE isoforms **(A)** and all isoforms **(B)** from the simulated data. Left panels: observed area under the ROC curve (AUC) for all methods; each method has 50 replicates. Right panels: the corresponding ROC curves averaged over 50 replicates.

Performances of the DE methods on the simulated data with the alternative hypothesis are also evaluated using the area under the ROC curve (AUC). In **Figure 4**, the AUC and ROC curves of top 5% DE isoforms and all isoforms over 50 replicates are presented in panels A and B, respectively. For edgeR and monocle, there are obvious differences between their performances for top 5% DE isoforms and for all isoforms. For the top 5% isoforms, these two methods perform poorly compared to the other methods. However, if all isoforms are considered, the two methods are comparable with the other methods when more isoforms are taken into account. Results for the top 10% and 20% DE isoforms are given in **Figure S3** in the Supplementary report. Among these methods, BPSC and DESeq2 are consistently the top performing methods with the highest AUC values for different sizes of top DE isoform sets. Overall, these results are in agreement with the results from RDR analyses.

## Materials and Methods

### Experimental and Synthetic Data Sets

To capture the true distributional characteristics of real data, three real scRNA-seq data sets are used for the evaluation of the nine DE methods. The first data set (MDA-MB-231) includes 160 single cells from a triple-negative breast cancer cell line, half of which are treated with metformin. The cells are captured using the Fluidigm C1 system and sequenced on Illumina HiSeq 2500 machines for 80 control and 80 treated cells separately. Then we use Cufflinks (Trapnell et al., 2010) to estimate the isoform expression. This data set contains a total of 26,775 isoforms across 160 single cells. The average number of reads per cell is ∼649,000.

The second data set (mESCs) is collected from a public scRNA-seq data (GSE60749-GPL13112) in the *Conquer* data set (Soneson and Robinson, 2018), which provides expression estimates of isoforms. The compared single cells are 94 individual v6.5 mouse embryonic stem cells (mESCs) with culture conditions 2i+LIF (group 1) vs. 174 v6.5 mESCs with culture conditions in serum+LIF (group 2). The data are prepared with the C1 System using the SMARTer Ultra Low RNA kit for Illumina Sequencing (Clontech) and protocols provided by Fluidigm. More details of the data can be found in the original paper (Kumar et al., 2014). Then the *Conquer* pipeline estimates isoform abundances using Salmon (Patro et al., 2017). This data set contains 112,593 isoforms across 174 single cells in group 1 and 94 single cells in group 2. The average number of reads per cell is ∼1.7M, the largest among the 3 data sets.

The third real data set (NPCs) is a subset of GSE102934 data from the NCBI Gene Expression Omnibus (Iacono et al., 2018). This data set has 720 NPCs derived from induced pluripotent stem (iPS) cells, half of which are from a Williams-Beuren patient and the other half are from a healthy donor. The data are sequenced on Illumina HiSeq 2500 platform and then applied massively parallel single-cell RNA sequencing (MARS-Seq) to construct single-cell libraries. This data set contains a total of 41,020 isoforms from 720 single cell, and the average number of reads per cell is 18,600. Thus, this data set has a relatively large number of cells with low sequencing coverage.

The simulated data set for isoform expression of single cells is generated by the beta-Poisson model (Vu et al., 2016). In particular, we generate the counts for each isoform from a beta-Poisson distribution with four parameters estimated from the mESCs data set. The four-parameter beta-Poisson model is as follows:

(1) $$BP_{4}(x|\alpha ,\beta ,\lambda_{1},\lambda_{2})=\lambda_{2}\text{Poison}(x|\lambda_{1}\text{Beta}(\alpha ,\beta ))$$

The mean and variance of the model can be written as

$$\mu =E(X)=\lambda_{1}\lambda_{2}\phi_{1}$$

and

$$V\;ar(X)=\mu \lambda_{2}+\mu^{2}\phi_{2},$$

where $\phi_{1}=\frac{\alpha }{\alpha +\beta }$ and $\phi_{2}=\frac{\beta }{\alpha (\alpha +\beta +1)}$. Crucially, we can modify the parameter λ_1_ to create mean differences between groups. A more detailed description of the model can be referred to in the original study (Vu et al., 2016).

Beta-Poisson models fitted on the real mESCs data set are used as baseline distributions for simulation. For each isoform, expression values across samples in the control and the treated group are generated from the same beta-Poisson model. To mimic the biological variation, 5% of isoforms are selected to be differentially expressed between two groups (true DE isoforms). Specifically, the parameter λ_1_, which controls the mean of the distribution, is fixed in the control group and multiplied by log_2_ fold change of 1 unit in the treated group. The effect direction is randomly determined for each DE isoform, with equal probability of upregulation and downregulation. In other words, the quantity change between the two compared groups is either two- or half-fold change with equal probability. The simulated data set consists of 80 samples in each of control and treated groups and a total of 10,000 isoforms measured per sample. Library sizes of the single-cell samples are randomly sampled from a range of 1–3 million. We filter out isoforms with zero expression across all samples.

### DE Analysis Methods

Nine DE methods included in this study are categorized into four groups based on different statistical models. These nine methods are selected to cover most statistical models used in recent DE analysis. Regarding other DE methods that are not included in this study, they use similar approach comparing to the nine selected methods. For instance, D3E (Delmans and Hemberg, 2016) utilizes beta-Poisson model which is similar to BPSC; SCDE (Kharchenko et al., 2014) models the gene expression values using a mixture of negative-binomial distribution for amplification components and a Possion distribution for dropout events, which is similar to DEsingle; Ballgown (Frazee et al., 2015) is based on the linear modeling strategy which is similar to limma. In this section, we give a brief summary of these nine methods. For more details of the software packages and statistical models, the reader is referred to original publications and related software websites. When applying these tools, we follow standard procedures and parameter settings suggested in software manuals.

#### Negative-Binomial–Based Methods

The read counts of an isoform from the technical replicates (repeated sequencing runs of the same sample) are usually modeled to follow a Poisson law (Marioni et al., 2008). However, those from the biological replicates are usually assumed to follow a gamma distribution to accommodate the overdispersion observed in empirical data (Chen et al., 2014). Since the negative binomial (NB) model can be derived as a gamma-Poisson mixture model, several DE methods based on the NB distribution assumption have been developed to accommodate the overdispersion among biological replicates. Note, however, that these theoretical motivations come from bulk-cell RNA-seq data. Two popular methods for this class are edgeR (Robinson et al., 2010) and DESeq2 (Love et al., 2014). The setup is then to assume the expression read counts *y_ij_* ~ *NB*(µ*_ij_*,φ*_i_*), where µ*_ij_* is the mean and φ*_i_* is the dispersion parameter for isoform *i* and sample *j*. Reliable estimation of the dispersion parameter φ*_i_* for each isoform is crucial for detecting DE isoforms. Differences in the estimation of φ*_i_* explain the main differences between edgeR and DESeq2.

##### edgeR

A conditional maximum likelihood (CML) is used in edgeR (Robinson et al., 2010) to estimate a common dispersion, which is assumed to be the same for all isoforms. Then this procedure is developed further to allow for the isoform-specific dispersion estimates and an empirical Bayes procedure—approximated by a weighted likelihood—is used to shrink the dispersions toward the common dispersion. The amount of shrinkage is determined by the neighbourhood set that is nearest to isoform *i* in average log count-per-million (logCPM). For DE testing, edgeR allows the user to select among different hypothesis tests including quasi-likelihood F-test (edgeRQLF) for bulk-cell RNA-seq data and likelihood ratio test (edgeRLFT) for scRNA-seq data as suggested by the developer. However, a recent study (Soneson and Robinson, 2018) shows that edgeRQLF performs significantly better than edgeRLFT in scRNA-seq data. Therefore, in this study, we report the results of edgeRQLF for the evaluation of edgeR in DE analysis.

##### DESeq2

DESeq2 (Love et al., 2014) uses a similar negative-binomial model as edgeR but facilitates more data-driven shrinkage estimators for dispersion and fold change. DESeq2 assumes the isoforms of similar average expression levels have similar dispersion and shrinks the isoform-specific dispersion toward a fitted smooth curve by an empirical Bayes approach. To overcome the difficulty in the log fold-change (LFC) estimation for the lowly expressed isoforms, DESeq2 shrinks LFC estimates toward zero when the expression level is low. The shrinkage procedure may result in underestimates of dispersion, thereby producing conservative estimate statistics for the DE test. This helps reduce the FPR at the expense of lower sensitivity.

##### DEsingle

DEsingle (Miao et al., 2018) has another negative-binomial based approach that employs the zero-inflated NB (ZINB) model to discriminate the observed zero values into two parts—constant zeros and zeros from the NB distribution. With the model, DEsingle is designed to overcome the issues of the excessive zero values observed in scRNA-seq data. To detect DE isoforms between two groups, DEsingle first calculates the maximum likelihood estimates (MLE) of two ZINB populations’ parameters, then computes the constrained MLE of the two models’ parameters under the null hypothesis (*H*_0_), and finally uses the likelihood ratio test for testing *H*_0_.

#### Beta-Poisson–Based Methods

##### BPSC

BPSC (Vu et al., 2016) is an analytical procedure based on the beta-Poisson mixture model, which is designed to capture the property of scRNA-seq data. The model is integrated into the generalized linear model (GLM) framework for DE analysis. The sophisticated four-parameter beta-Poisson model is as shown in Eq. (1). The iterative weighted least-squares (IWLS) algorithm is used to estimate the model parameters.

#### Normal-Based Methods

##### Limma

Limma (Law et al., 2014) method is based on linear modelling which was originally designed for gene expression microarray data, but has recently been extended to RNA-seq data. In this study, we use limmatrend (Law et al., 2014), a version of limma where the empirical Bayes procedure is modified to incorporate a mean-variance trend for DE analysis. In a recent study of DE analysis of scRNA-seq data (Soneson and Robinson, 2018), limmatrend has the best performances among other versions of limma, such as voomlimma.

##### Monocle

Monocle (Qiu et al., 2017) is a tool originally designed for scRNA-seq data for identifying DE genes that vary across different cell types or across a so-called “pseudo-time.” The mean expression level of each isoform is modeled by generalized additive models (GAMs) which relate one or more predictor variables to a response variable as

$$g(E(Y))=\beta_{0}+f_{1}(x_{1})+f_{2}(x_{2})+\cdot \cdot \cdot +f_{m}(x_{m}),$$

where *Y* is a response variable, and *x_i_*‘s are predictor variables. The function *g* is a link function, typically the log function, and *f_i_*‘s are nonparametric functions, such as cubic splines or other smoothing functions. Gene expression level across cells is modeled by a Tobit model; with some approximations, monocle’s GAM is thus

$$E(Y)=s(\psi_{t}(b_{x},s_{i}))+\epsilon ,$$

where ψ_t_(*b_x_*,*s_i_*) is the assigned pseudo-time of a cell and *s* is a cubic smoothing function with (by default) three effective degrees of freedom. ϵ is the error term that is normally distributed with a mean of zero. The DE test is performed with a *x* ^2^-approximation of the likelihood ratio test.

##### MAST

MAST (Finak et al., 2015) uses a hurdle model tailored to scRNA-seq data. It is a two-part GLM that simultaneously models the gene expression rate (how many cells express the gene) by logistic regression and the expression level by Gaussian distribution. The DE testing is then done using the likelihood ratio test.

##### T-Test

T-test (Welch, 1947) is a general comparison method that is used to compare the means of two groups. One of the most common assumptions made when doing a t-test is the normality of data distribution. Empirically, scRNA-seq data are highly skewed, but the t-test is known to have a certain robustness against skewness, so it is still worth comparing against other sophisticated methods.

#### Nonparametric Methods

##### Wilcoxon Rank Sum Test

Wilcoxon rank sum test (Hollander et al., 2013) (also known as Mann-Whitney test) is a nonparametric test that is used to determine whether the two independent samples come from the same distribution. The main idea of the test is to compare the sum of the ranks for the observations which come from different samples.

## Discussion

We have performed a systematic comparison of nine different statistical methods for DE analysis of scRNA-seq data. To get realistic distributional characteristics, three real scRNA-seq data sets are used as the basis for generating the data. A beta-Poisson model–based simulated data set is also performed to assess the performance of each method. The nine methods are evaluated by the type-I error control, the ROC curve and the RDR under both null and alternative hypotheses. Our results show that lowly expressed isoforms are generally the source of strong differences between methods. Most methods except monocle have good RDR performances for highly expressed isoforms.

EdgeR and monocle tend to produce extremely small p-values for lowly expressed isoforms, leading to many false positives. Notably, these two methods perform very poorly compared to the other methods for top DE isoforms. These results are consistent with other recent studies(Dal Molin et al., 2017; Soneson and Robinson, 2018). DESeq2, a bulk-cell–based method with a shrinkage procedure, works rather well over all isoforms on both the real scRNA-seq data and the simulated data. However, DESeq2 is highly conservative for lowly expressed isoforms, so its sensitivity is always lower than the other methods for all three real data sets. The performances of BPSC are comparable to DESeq2 in all analyses but less conservative. Other methods including limmatrend, t-test, Wilcoxon, MAST, and DEsingle perform reasonably in both real and simulated data sets.

To validate our results, we analyzed two extra public real scRNA-seq data sets including one data set with 164 single cells from H7 human cell-line generated by the SMARTer C1 prototol and another big data set contain 2,027 intestinal single cells of mouse from the CEL-Seq protocol. The results in **Figure S6-S8** show the consistency of the comparison analyses for different types of scRNA-seq data for the new small data set. But for the new big data set, monocle and DESeq2 show particularly low sensitivity for lowly expressed isoforms in **Figure S6D-S8D**. The details of these data and results are referred to the **Supplementary Material**.

We also investigated further the performances of the DE methods for the group of lowly expressed isoforms. We first checked the relationship between the performance of the Wilcoxon test, one of the most stable DE methods, and the signal strength in different log fold-change (LFC) 1, 2, 3, and 4 using the simulated dataset. Results in the **Figure S4** show that the RDR of Wilcoxon is a function of signal strength where it achieves a higher RDR for the data with a higher LFC. The low signal in the simulated data in **Figure 3D** had made the differences of true RDR for different methods inconspicuous. So we generated another simulation data set using the same procedure described in 3.1 but with a high signal strength LFC = ± 4, then applied all 9 methods on the simulated lowly expressed genes. The results (**Figure S5**) confirmed that for the lowly expressed isoforms, DESeq2 is too conservative and consequently loses sensitivity compared to the other methods.

The nine methods compared in this study are selected to cover most statistical models used in recent DE analysis. Although some DE methods are not included in this study, they use similar approach to those we included. For instance, D3E (Delmans and Hemberg, 2016) utilizes beta-Poisson model which is similar to BPSC; SCDE (Kharchenko et al., 2014) models the gene expression values using a mixture of NB distribution for amplification components and a Possion distribution for dropout events, which is similar to DEsingle; Ballgown (Frazee et al., 2015) is based on the linear modeling strategy which is similar to limma.

The main strengths of our comparison method include (i) the use of three real scRNA-seq data sets in order to capture the true distributional characteristics and the diversity of single-cell data; (ii) the use of the RDR metric for top-rank genes. This is consistent with the data analysis process of identifying the list of interesting genes. In some cases we show that considering the full collection of genes will lead to misleading comparisons; (iii) Separate results of highly and lowly expressed genes, as these two groups have distinct distributions and the methods vary more in their performances for lowly expressed genes. In summary, performances of DE methods do vary, so we need to pay attention in choosing the method to use, and, at least for highly expressed genes, some methods designed for bulk-cell RNA-seq analysis do not necessarily perform worse than those specifically designed for scRNA-seq data. Finally, as shown the figures, the number of lowly expressed genes is not trivial, so our results also highlight the need for further development of methods to deal with these genes.

## Conclusion

There are large differences in the performance of methods for detecting DE in single-cell RNA-seq data. This is driven partly by the expression level of genes. For highly expressed genes, many bulk-cell–based DE methods perform well against single-cell–based methods. But, for lowly expressed genes, the performance of the methods varies, so a careful check of the gene expression level should be made before choosing a DE method in analyses. This is to ensure that the chosen method is appropriate for your data. We found edgeR and monocle to have poor control of false-positives on lowly expressed genes, so we do not recommend these two methods for such genes. DESeq2 tends to be too conservative, so it sacrifices sensitivity for higher specificity. According to the simulation results, BPSC performs well against the other methods, particularly when there is a sufficient number of cells. RDR for top-rank genes is a useful metric for assessing performance of DE methods, sometimes giving different results compared to analysis of the full set of genes. We suggest to be considered in choosing DE methods to use, performances of DE methods in scRNA-seq data strongly depend on the expression level of genes.

## Data Availability Statement

The raw data of two data sets mESC (GSE60749-GPL13112) and NPCs (GSE102934) are published by the original studies and publicly available from NCBI Gene Expression Omnibus repository. The gene expression data of these data sets, MDA-MB-231 data set, simulated data sets and the two supplementary data sets can be found at: https://github.com/Tianmou/scRNAseq-DE-comparison.

## Author Contributions

YP and TM designed the study. TM, TV, and YP performed the analysis and wrote the manuscript. WD performed the acquisition of MDA-MB-231 data. FG performed a part of simulation studies. All authors read and approved the final manuscript.

## Funding

This work was partially supported by funding from the Swedish Cancer Fonden, the Swedish Research Council (VR) and the Swedish Foundation for Strategic Research (SSF).

## Conflict of Interest

The authors declare that the research was conducted in the absence of any commercial or financial relationships that could be construed as a potential conflict of interest.
