# Supplementary material for: Reproducibility of Methods to Detect Differentially Expressed Genes from Single-Cell RNA Sequencing
Source: Front Genet. 2020 Jan 17;10:1331. doi: 10.3389/fgene.2019.01331 (PMC6979262; doi:10.3389/fgene.2019.01331)
Supplement: Supplementary file 1 [file DataSheet_1.pdf]

Supplementary Material

1 SUPPLEMENTARY FIGURES

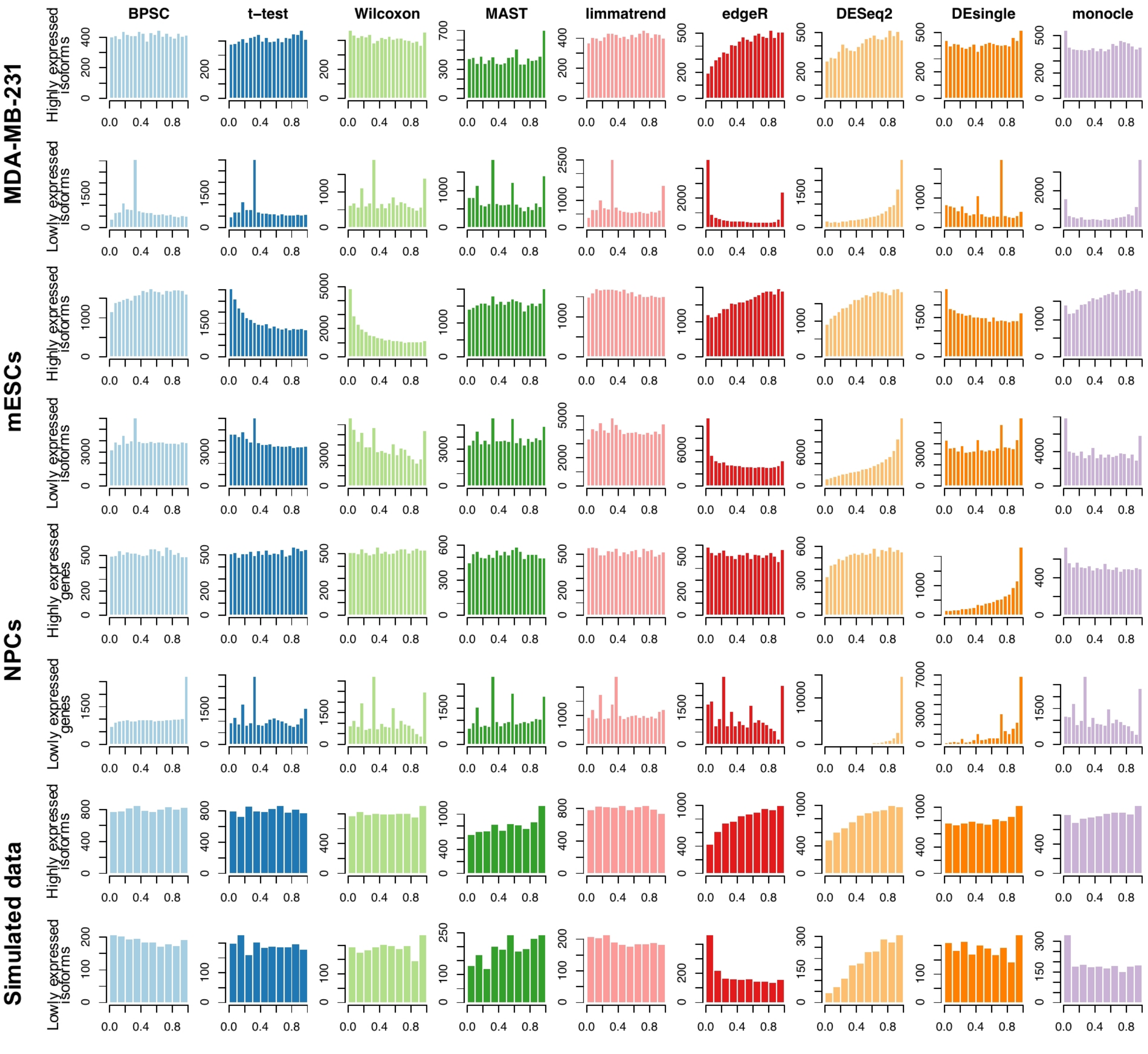

Figure S1. Histograms of P-values under the null hypothesis for all methods applied on three real data sets and BP simulated data set. The values in the plot are from one replicate.

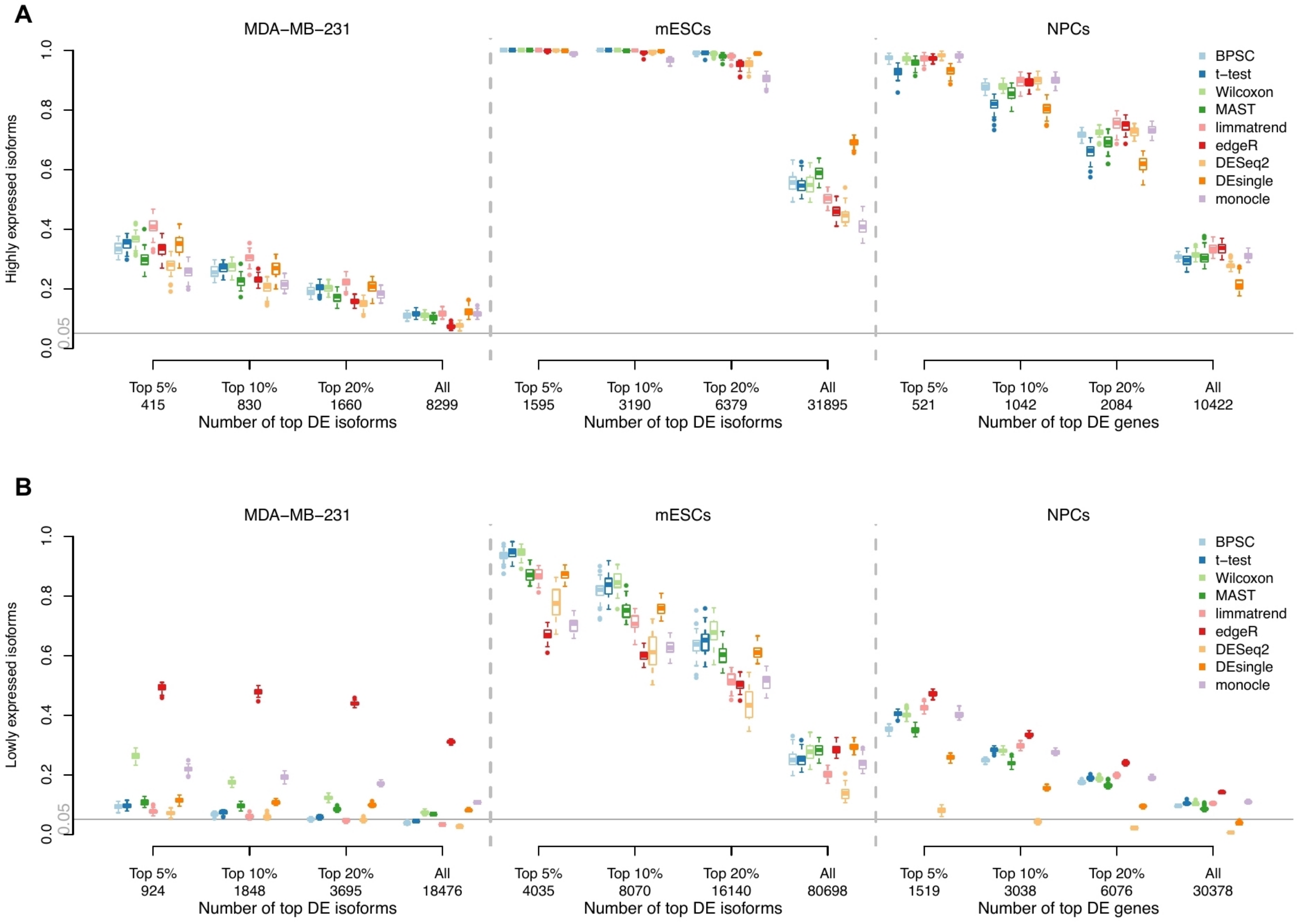

Figure S2. Rediscovery rate of DE isoforms in three real scRNA-seq datasets under the alternative hypothesis calculated among the top 5%, 10%, 20% DE isoforms (or genes for NPCs data set). The isoforms are splited into two groups based on expression levels. Highly expressed isoforms refer to the isoforms with an estimated expression above 1 TPM in more than 25% of the cells, and the rest are lowly expressed isoforms.

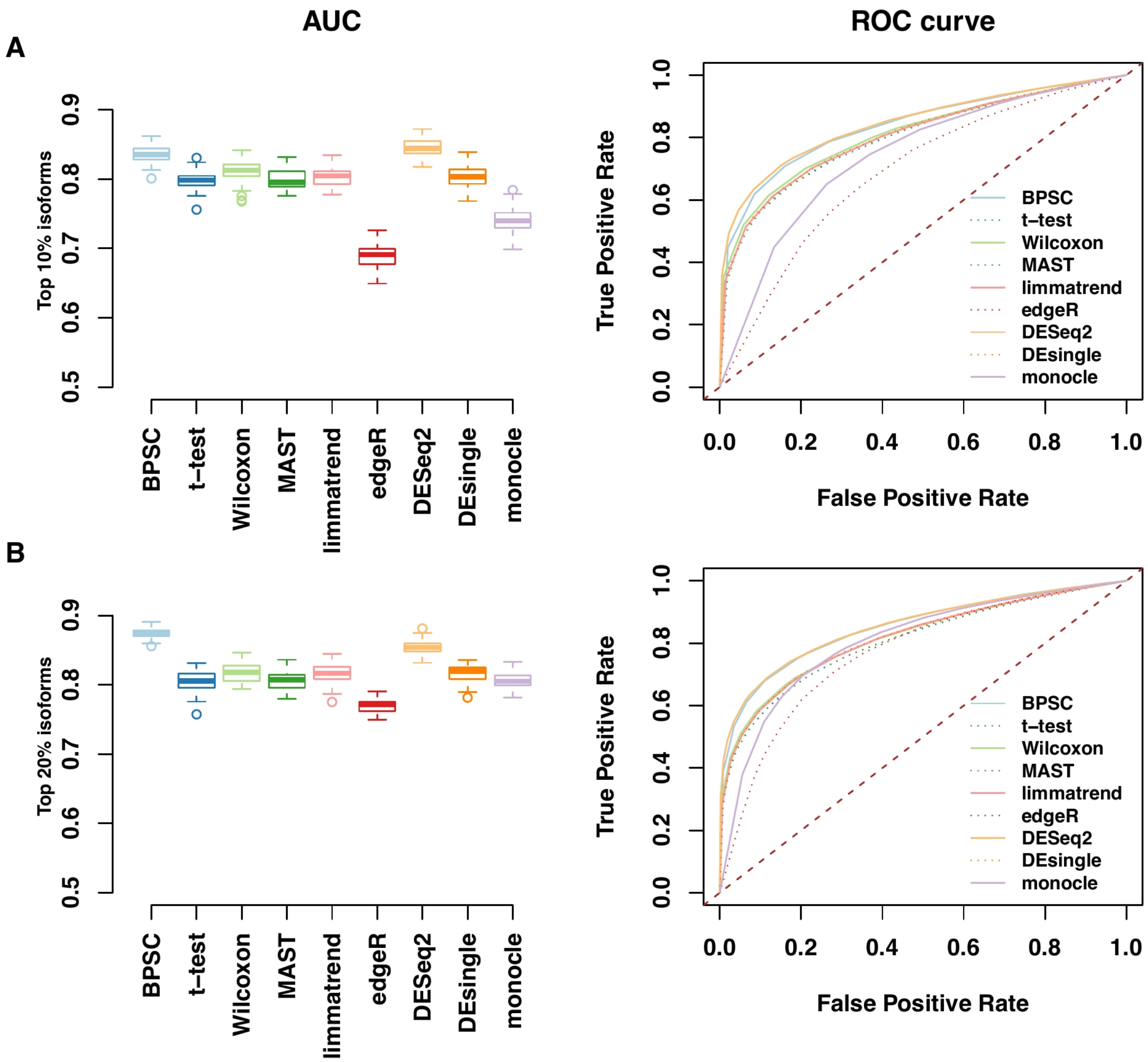

Figure S3. ROC and AUC performances for top 10% (A), 20% (B) DE isoforms from the simulated data. Left panels: observed area under the ROC curve (AUC) for all methods, each method has 50 replicates. Right panels: the corresponding ROC curves averaged over 50 replicates.

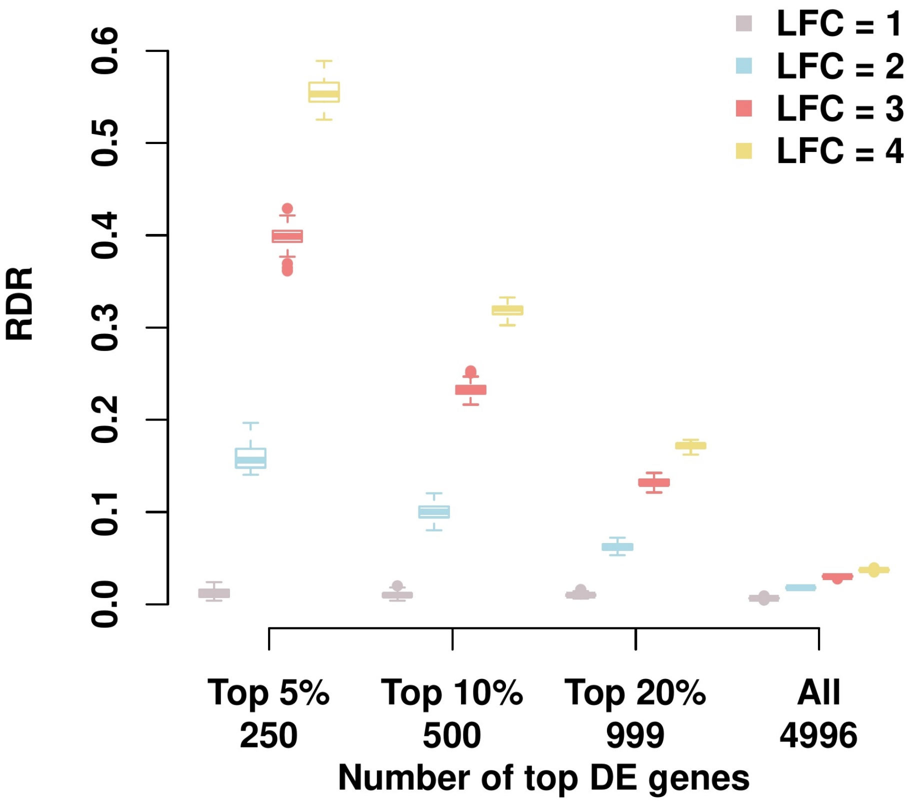

Figure S4. Rediscovery rate of Wilcoxon method in the simulated data with different signal strengths (LFC=1,2,3,4). The RDR is calculated based on lowly expressed isoforms.

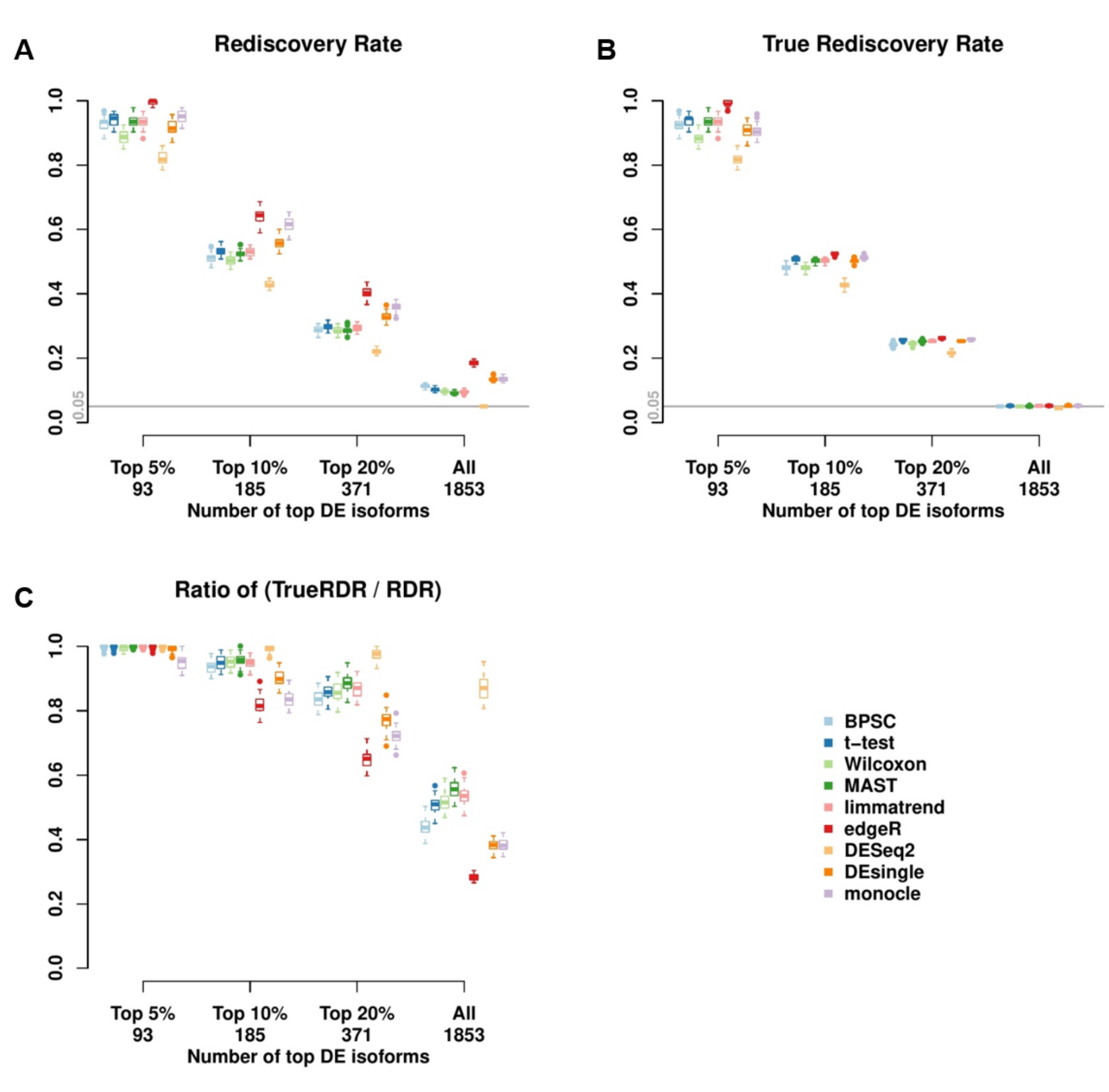

Figure S5. Results comparing 9 methods for the simulated data with LFC = 4. (A) Rediscovery rate (B) true rediscovery rate (C) ratio of true RDR / RDR under the alternative hypothesis. The statistics are calculated based on lowly expressed isoforms.

2 ANALYSES FOR TWO SUPPLEMENTARY scRNA-Seq DATA SETS

We further extended the analyses the DE methods for two more public scRNA-seq datasets: 1) SRP073808 [PMID: 31524596], a small data set from human contains 77 in vitro cultured H7 embryonic stem cells (WiCell) and 87 cells from H7-derived downstream early mesoderm progenitors. The sequencing for this dataset is implemented using the SMARTer C1 protocols with average number of reads per cell is around 1.5M; and 2) GSE62270 [PMID: 26287467], a big dataset includes 1344 cells from mouse intestinal organoids and 683 Reg4-positive intestinal cells. Each single cell contains in average 9,689 reads generated using the CEL-Seq protocol. The gene expression of these data sets used in this study are collected from the Conquer project [PMID:29481549]. The results in figures S6-S8 show the consistency of the comparison analyses for different types of scRNA-seq data for the new small data set SRP073808. But for the new big data set GSE62270, monocle and DESeq2 show particularly low sensitivity for lowly expressed isoforms in figure S6-D, S7-D and S8-D.

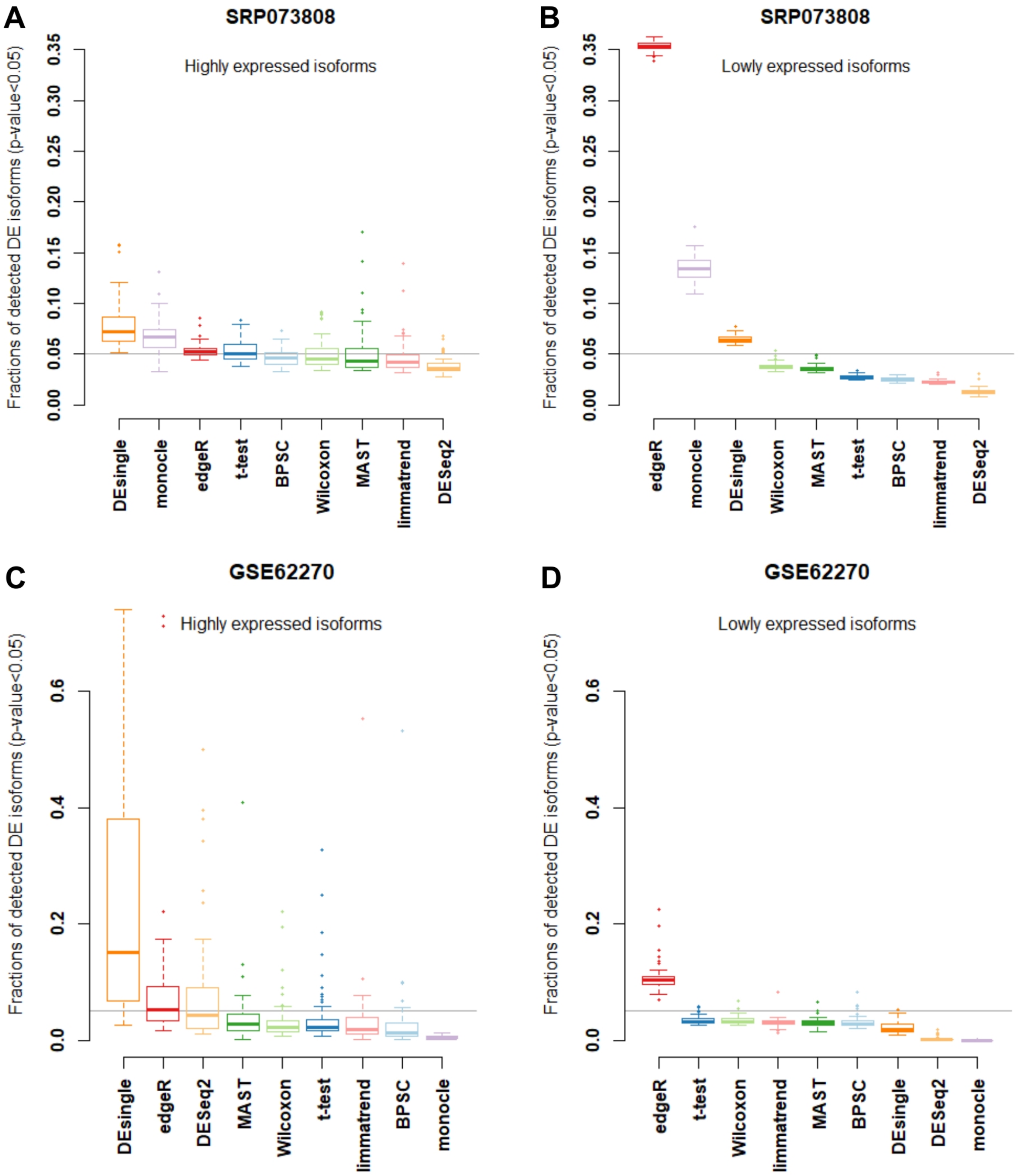

Figure S6. Type-I error of the two supplementary scRNA-seq data sets. The isoforms are splited by highly (left) and lowly (right) expressions. The values in the y-axis are the fractions of isoforms with $p < 0.05$ under the null hypothesis. The horizontal line indicates the expected error rate at 0.05. Box plots of the methods in the x-axis are the collection from 50 replicates. The methods are ordered by median FPR across all replicates. The number of highly expressed isoforms in SRP073808 is 24,068. The corresponding number of lowly expressed isoforms is 192,355.

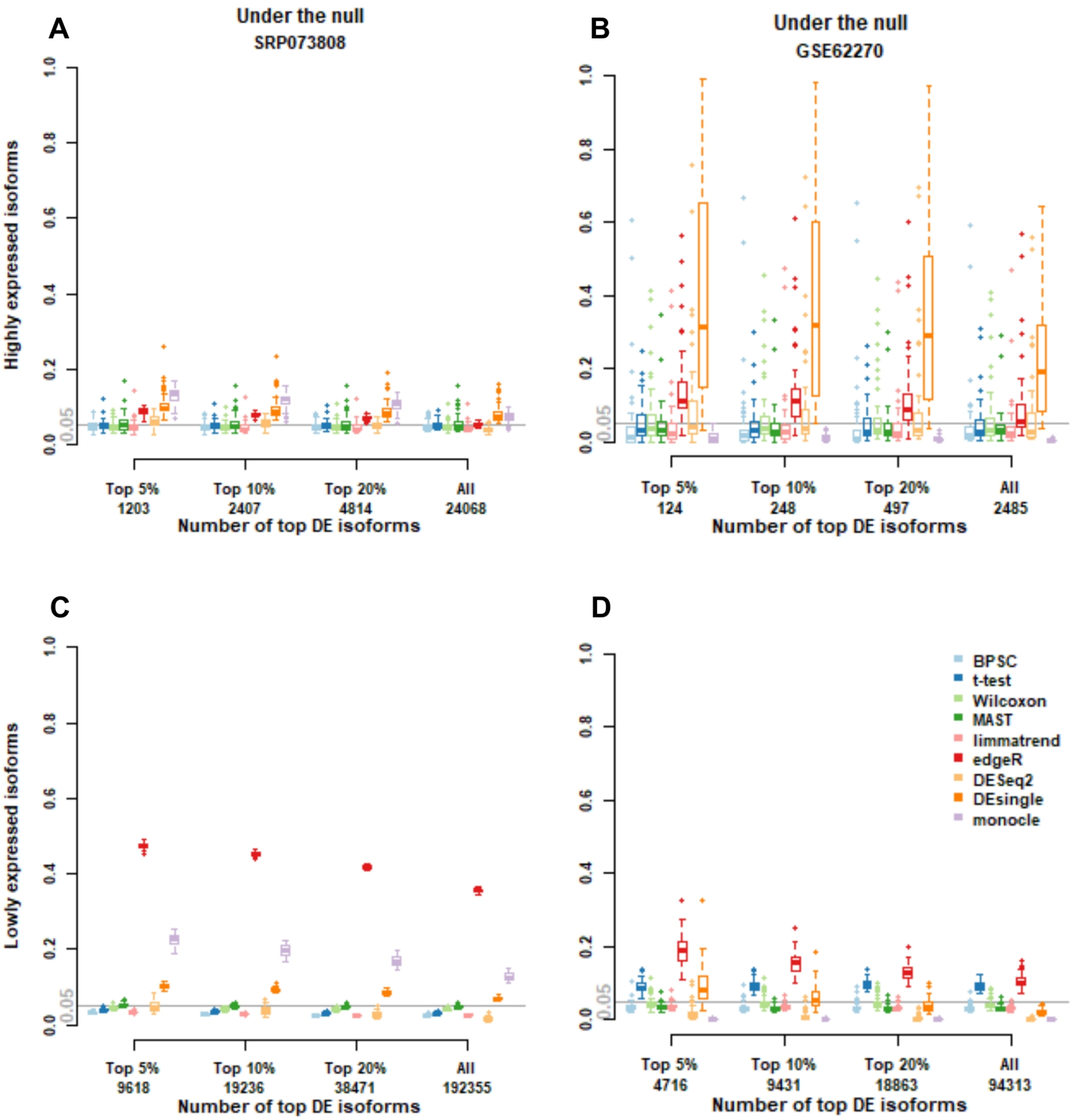

Figure S7. Rediscovery rate of DE isoforms in the two supplementary scRNA-seq data sets under the null hypothesis calculated among the top 5%, 10%, 20% DE isoforms. The isoforms are splited into two groups based on expression levels. Highly expressed isoforms refer to the isoforms with an estimated expression above 1 TPM in more than 25% of the cells, and the rest are lowly expressed isoforms. A and C are from SRP073808, B and D are from GSE62270.

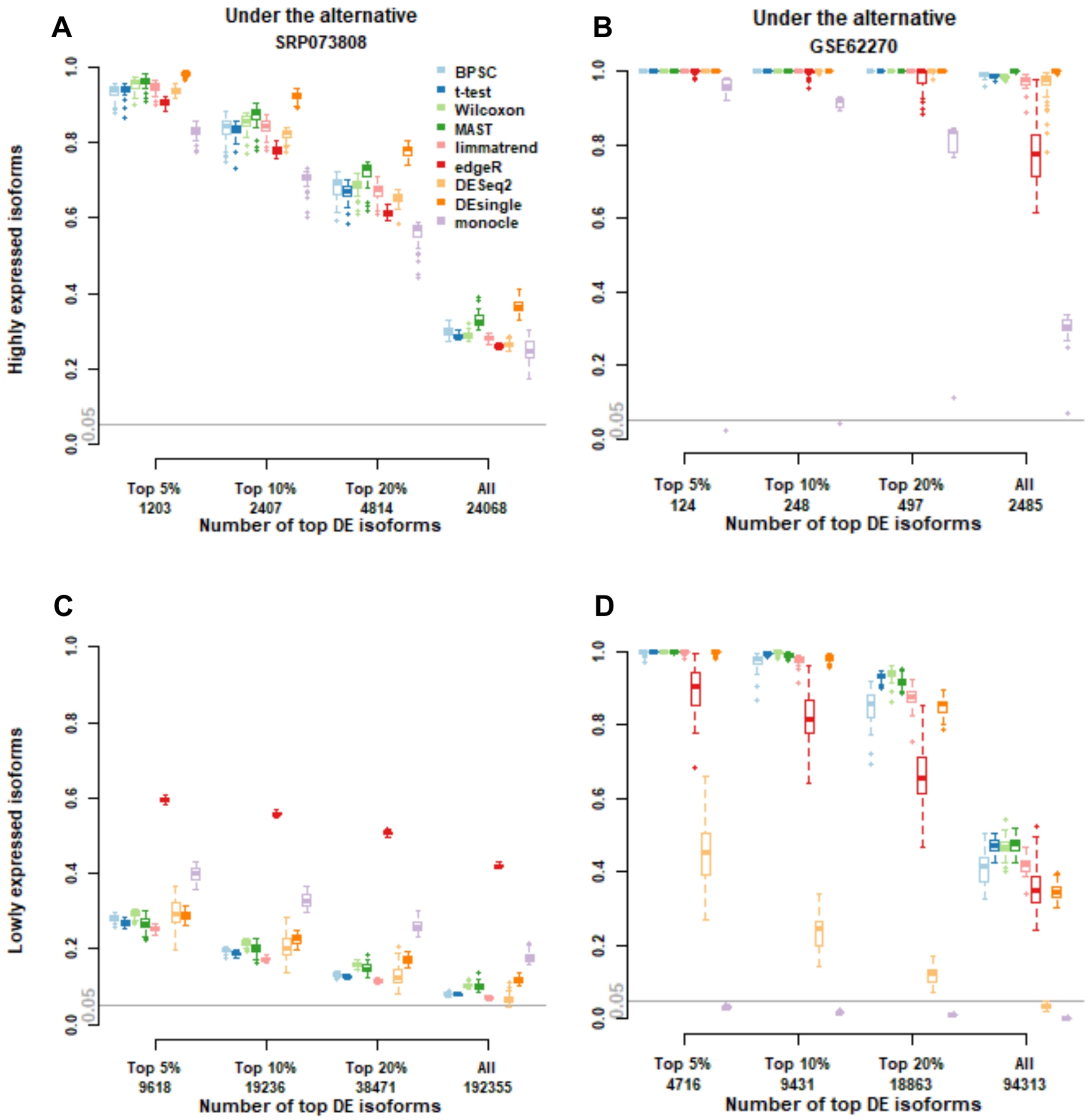

Figure S8. Rediscovery rate of DE isoforms in the two supplementary scRNA-seq data sets under the alternative hypothesis calculated among the top 5%, 10%, 20% DE isoforms. The isoforms are splited into two groups based on expression levels. Highly expressed isoforms refer to the isoforms with an estimated expression above 1 TPM in more than 25% of the cells, and the rest are lowly expressed isoforms. A and C are from SRP073808, B and D are from GSE62270.
